# College students’ in Jamaica opinion about older adults and their role as future caregivers: Case Example

**DOI:** 10.1093/geroni/igab046.2791

**Published:** 2021-12-17

**Authors:** Sharon McKenzie, Sandra Latibeaudiere

**Affiliations:** 1 Kean University, Union, New Jersey, United States; 2 University of the West Indies, Kingston, Kingston, Jamaica

## Abstract

Ageing is a natural human experience, yet for many people getting old provoke fears of vulnerability, fragility, undesirability, and incapacity. Such perceptions are often attributed to various taboos, prejudices, discrimination and stereotype associated with old age. The purpose of this study was to assess college students’ in Jamaica opinion about older adults and their role as future caregivers. Specifically, the study sought to: (1) identify whether college students hold ageist stereotype/negative image about older adults and whether they influence future role as caregiver, 2) assess their knowledge of the chronic conditions that affect older adults, and 3) assess whether they see themselves working or taking an active role in future caregiving. As we think about the future directions of healthcare provision for older adults, this small sample of college students provided a discourse about ageing and key elements that are important for educators in a developing country such as Jamaica to consider when building a gerontology curriculum.

